# Catecholamines in neuroblastoma: Driver of hypertension, or solely a marker of disease?

**DOI:** 10.1002/cnr2.1569

**Published:** 2021-10-06

**Authors:** Matthew Harding, Rebecca J. Deyell, Tom Blydt‐Hansen

**Affiliations:** ^1^ Division of Nephrology, Department of Pediatrics British Columbia Children's Hospital Vancouver British Columbia Canada; ^2^ Division of Pediatric Hematology/Oncology, Department of Pediatrics British Columbia Children's Hospital Vancouver British Columbia Canada

**Keywords:** catecholamine, hypertension, metanephrine, neuroblastoma

## Abstract

**Background:**

Neuroblastoma is a common solid tumor of childhood and is often associated with hypertension. Potential etiologies contributing to hypertension include renal compression, pain, volume overload, and catecholamine secretion.

**Cases:**

We completed a single center retrospective review of children with neuroblastoma and ≥stage II hypertension (per Hypertension Canada guidelines) over a 2‐year period. All patients (*n* = 10) had elevated urine normetanephrine levels and eight had intra‐abdominal tumors. Four patients had refractory hypertension requiring > three agents, of which three required alpha/beta blockade.

**Conclusion:**

Although multifactorial, hypertension in neuroblastoma often has a neuroendocrine component. Excess normetanephrine production in neuroblastoma may be a more common hypertensive mechanism than previously appreciated. Urinary normetanephrine elevation could suggest potential neuroendocrine‐mediated hypertension.

## 1 INTRODUCTION

Neuroblastoma is the most common extracranial solid tumor of infancy.^1^ Tumors arise from the post‐ganglionic sympathetic nervous system and often present with a suprarenal mass. Patients with high‐risk disease often present with irritability, pain, cytopenias, and fever.^2^, ^3^

A number of reports describe severe hypertension associated with neuroblastoma.^2^, ^4^, ^5^, ^6^, ^7^, ^8^, ^9^, ^10^, ^11^ Patients may have transient hypertension, which can be exacerbated by induction of anesthesia or intraoperative tumor manipulation.^6^, ^7^, ^8^, ^9^, ^10^, ^11^ Others may develop refractory hypertension around the time of administration of chemotherapy. Therapeutic ^131^I‐metaiodobenzylguanidine (MIBG) can also precipitate hypertension.^10^ Hypertension is often attributed to renin‐mediated effect when renal artery or parenchymal compression is present.^11^, ^12^, ^13^ Obstruction of urinary flow may also result in hypertension. Marrow involvement can cause pain and chemotherapy treatment protocols often include hyperhydration, both of which may exacerbate hypertension.^14^, ^15^

Excess catecholamine production in neuroblastoma can directly elevate blood pressure (BP).^16^ Urinary vanillylmandelic acid (VMA) and homovanillic acid (HVA) levels are routinely assessed at the diagnosis of neuroblastoma and are elevated in 90%–95% of patients.^17^, ^18^ However, baseline normetanephrine levels are not consistently assessed in standard protocols, despite being excreted by neuroblastoma and being associated with hypertension.^16^, ^19^ We review the etiology, management and outcome of neuroblastoma‐associated hypertension in a cohort of pediatric patients.

## 2 METHODS

Following research ethics board approval, we identified all children (<18 years) diagnosed with neuroblastoma and stage II hypertension (per Hypertension Canada guidelines) treated at a tertiary care pediatric center over 2 years (2015–17).^20^ Cases were reviewed retrospectively. No explicit exclusion criteria were used. Collected data included demographics, tumor location/INRG stage, evidence for renal compression and Children's Oncology Group (COG) risk group, as well as hypertension severity, sequelae and duration. Maximal systolic blood pressure (*n* = 9; SBP) or maximal mean arterial pressure in a neonate (*n* = 1; MAP) was reported as Z‐score for height, sex, and age.^21^, ^22^ Patients were reported as hypertensive with BP >95th centile on ≥ two occasions in each 24‐h period. Estimated glomerular filtration rate (eGFR) was calculated via Schwartz formula.^23^

Spot urine VMA, HVA, and normetanephrines were collected at baseline for all patients, and reported referenced to the upper limit of reference range (ULRR). The majority (70%) had urine metanephrines collected concurrently. Thereafter, catecholamine surveillance was variable; some patients had no further monitoring.

## 3 RESULTS

Median age at neuroblastoma diagnosis was 2.1 years (range: 10 days–8.5 years). Four patients were female and five had high‐risk disease (*MYCN* amplified in one patient). (Table 1) The primary tumor was intra‐abdominal in eight patients; four had stage M and one had MS disease. SBP/MAP median Z‐score at presentation was 3.5 (range: 2.7–5.6). Maximal SBP/MAP median Z‐score was 4.7 (range: 2.8–10.3). No patient was on prior antihypertensive therapy. All but one patient had normal GFR at diagnosis (minimum: 21 ml/min/1.73 m^2^). Volume overload was observed in 40% of cases during the first week in hospital.

**TABLE 1:** Patient characteristics at diagnosis

Patient ID	Age	COG risk classification	INRG stage^24^	N‐Myc status	Lateralization/Location	Renal capsule or vascular involvement	Adrenal involvement	Paraspinal chain involvement	SBP^a^ percentile	DBP percentile	eGFR (ml/min/1.73 m^2^)	Volume overload during initial presentation (%)
1	5–18 months	Low	MS	NA	Right sided, above diaphragm	N	N	N	>99	85.8	94	6
2	>5 years	High	M	NA	Right sided, suprarenal	N	Y	Y	>99	69.2	106.5	0
3	<5 months	Low	L1	NA	Right sided, suprarenal	Y	Y	N	>99	‐	21	0
4	5–18 months	Intermediate	L2	NA	Right sided, above diaphragm	N	N	N	>99	>99	109.5	5
5	5–18 moths	Intermediate	L2	NA	Non‐lateralized, retroperitoneal	Y	Y	N	>99	98.4	105.4	0
6	>5 years	High	M	NA	Left sided, suprarenal	N	Y	N	>99	59.9	162.4	6
7	18 months–5 years	High	M	NA	Left sided, above diaphragm	N	Y	Y	>99	38.3	110	0
8	18 months–5 years	High	M	Amp	Non‐lateralized, retroperitoneal	Y	Y	Y	>99	>99	108.9	16
9	>5 years	Low	L1	NA	Right sided, retroperitoneal	N	N	N	>99	53.5	116.2	0
10	18 months–5 years	High	M	NA	Non‐lateralized, Retroperitoneal	Y	Y	Y	>99	>99	110.7	0

Abbreviations: Amp, amplified; COG, Children's Oncology Group; DBP, diastolic blood pressure; eGFR, estimated glomerular filtration rate; INRG, International Neuroblastoma Risk Group; MAP, mean arterial pressure; mmHg, millimeters of mercury; NA, non‐amplified; SBP, systolic blood pressure.

^a^ Mean arterial pressure used in one patient (antenatal diagnosis).

All patients had elevation of urinary VMA/creatinine ratio (median: 4.3 × ULRR, range: 0.8–20.9), HVA/creatinine ratio (median: 3.0 × ULRR, range: 1.4–16.3), and normetanephrine/creatinine ratio (median: 4.5 × ULRR, range: 1.4–140.1) at diagnosis (Table S1). Urinary metanephrine and epinephrine levels were normal, when measured. Renin was measured in four patients (40%) and was normal in all cases.

One of eight patients (12.5%) who received chemotherapy had >10 mmHg SBP increase in the 48 h following initiation of therapy. This patient was one of six who had hyperhydration for cyclophosphamide in cycle one. Three of nine patients who underwent tumor manipulation (33%) had >10 mmHg SBP increase in the 48 h following intervention; one post‐biopsy and two post‐tumor resection. No SBP increase was noted in the other six. One patient developed hypertension following anesthesia induction for imaging. One patient who was normotensive during tumor manipulation had prior alpha/beta blockade.

Seven patients required anti‐hypertensive treatment. (Table 2) Five required therapy for > 1 week, and four required > three medications to achieve normotension. For patients receiving alpha/beta blockade, BP normalized over a median of 26 days (range: 24–44 days). For this subset, resection was completed a median of 106 days after anti‐hypertensive initiation (range: 23–132 days) and a median of 16 days after alpha blockade initiation (range: 13–113 days). For one patient, discontinuation of antihypertensives resulted in recurrent hypertension.

**TABLE 2:** Evolution of hypertension; complications and treatment modalities

Patient ID	Onset of stage I HTN (days post diagnosis)	Onset of stage 2 HTN (days post diagnosis)	Initiation of treatment of HTN (days post diagnosis)	Total duration of treatment for HTN (days post initiation of therapy)	Time until control of HTN (days post initiation of therapy)	Medications used to control blood pressure (sequential)	Time started on chemotherapy (days post diagnosis)	Time of surgical resection (days post diagnosis)	Maximum volume overload (% greater than 100)	Development of AKI (days post diagnosis)	Severity of AKI (KDIGO stage)	Duration of AKI (days)	Evidence of end‐organ damage from HTN
1	5	5	N/A	N/A	N/A	N/A	9	N/A	8	N/A	N/A	N/A	None
2	0	0	N/A	N/A	N/A	N/A	8	93	0	2	1	1	None
3	0	3	1	8	8	Furosemide, hydralazine	N/A	N/A	18	1	1	1	None
4	0	0	N/A	N/A	N/A	N/A	15	69	5	N/A	N/A	N/A	None
5	0	0	2	Prazocin monotherapy at time of data collection	28	Nifedipine, amlodipine, hydralazine, **prazocin**, propranolol, atenolol, metoprolol	5	108	20	N/A	N/A	N/A	None
6	0	1	388	20	1	Furosemide, hydralazine	8	5	7	N/A	N/A	N/A	None
7	0	0	10	34	14	**Amlodipine**	9	N/A	7	134	2	1	Proteinuria on U/A
8	0	0	4	16	16	Furosemide, hydralazine, amlodipine, nifedipine	5	2	16	N/A	N/A	N/A	Proteinuria on U/A
9	0	0	1	24	26	Amlodipine, prazocin, nifedipine, **phenoxybenzamine**, atenolol, propranolol	N/A	24	0	N/A	N/A	N/A	Moderate LVH; resolved on repeat echo
10	0	0	1	133	66	Amlodipine, furosemide, nifedipine, spironolactone, captopril, phentolamine, **labetalol**, **phenoxybenzamine**, hydrochlorothiazide	9	133	17	19	2	8	Proteinuria on U_PCR_: 2.9 g/mmol Echo showed diastolic dysfunction

*Note*: Bolded anti‐hypertensive medications were titrated to achieve blood pressure control. In cases were multiple medications were used, the bolded medications required titration before control of hypertension was achieved.

Abbreviations: AKI, acute kidney injury; echo, echocardio; HTN, hypertension; KDIGO, Kidney Disease, Improving Global Outcomes; U/A, urinalysis; U_PCR_, urine protein to creatinine ratio.

Four patients had evidence of hypertensive end organ damage: two had proteinuria (detected 1 and 84 days after hypertension diagnosis), one had left ventricular hypertrophy (detected 1 day after hypertension diagnosis) and one developed acute kidney injury (18 days after hypertension diagnosis) with proteinuria and diastolic dysfunction. Proteinuria and cardiac dysfunction resolved over time in all cases.

## 4 DISCUSSION

This series describes the clinical course of patients with neuroblastoma and hypertension. All patients had elevation of urinary metanephrines which could suggest a component of neuroendocrine‐mediated hypertension. Although hypertension in neuroblastoma is viewed as multifactorial, other contributors to hypertension were not consistently identified.^11^, ^12^ Volume overload was present in one case, but did not precede onset of hypertension. No patient had an identified obstructive cause. Four patients had direct radiologic evidence of renal compression, although renin was normal in all patients in whom it was measured.

In patients with refractory hypertension (>3 agents required for control), the majority (80%) required alpha/beta blockade to achieve normotension. Alpha/beta blockade is employed in other catecholamine secreting tumors and in neuroblastoma in the peri‐operative context.^25^ This treatment approach mitigates alpha agonism on vascular smooth muscle and prevents “catecholamine storm” with tumor manipulation or anesthetic induction. Catecholamine‐induced hypertension has been previously described as rare in neuroblastoma, with hypertension attributed more commonly to renin‐mediated mechanisms in some case series.^11^, ^13^ In contrast, alpha/beta blockade was essential for hypertensive control of refractory hypertension in our series, suggesting that catecholamines play an important role in this patient subset.

There are several clinical implications to these data. Although urine VMA/HVA are routinely assessed at diagnosis of neuroblastoma, urine normetanephrines are not. In our study, patients with elevated urine normetanephrines often had hypertension that required targeted pharmacotherapy. Identification of elevated normetanephrines may guide hypertensive management, and urinary screening may be warranted when hypertension is identified at diagnosis. In cases with elevated urinary normetanephrines, alpha/beta blockade may be the preferred antihypertensive approach. End‐organ injury was evident in 40% of children with neuroblastoma‐related hypertension at diagnosis. Since prior hypertensive duration is difficult to estimate, proactive screening for end‐organ effects should be completed.

Our study has several limitations. Because our series reviewed patients with neuroblastoma and hypertension, our observations have limited generalizability for the neuroblastoma population without hypertension. Pain may have contributed for some patients, but retrospective review limited availability of this data. Hypertension severity was variable; lower stages of hypertension were not included. We cannot comment on whether patients with milder elevations in BP and elevated urinary normetanephrines are as likely to have a neuroendocrine mediated component to their presentations.

Excess normetanephrine production in neuroblastoma may be a more common hypertensive mechanism than previously appreciated. Urinary normetanephrine elevation should suggest potential neuroendocrine‐mediated hypertension. This has important implications for secondary hypertension risk stratification and management.

## CONFLICT OF INTEREST

The authors have stated explicitly that there are no conflicts of interest in connection with this article.

## AUTHOR CONTRIBUTIONS

**Matthew Harding:** Conceptualization (equal); data curation (lead); methodology (supporting); writing – original draft (lead); writing – review and editing (equal). **Rebecca J. Deyell:** Conceptualization (equal); data curation (supporting); methodology (equal); supervision (equal); writing – review and editing (equal). **Tom Blydt‐Hansen:** Conceptualization (equal); data curation (supporting); methodology (equal); supervision (equal); writing – review and editing (equal).

## ETHICS STATEMENT

This project received institutional ethics approval from the University of British Columbia Clinical Research Ethics Board; this study was performed in accordance with the ethical standards laid down by the 1964 Declaration of Helsinki.

## CONSENT STATEMENT

Prior informed consent was not obtained as the research ethics board determined this project met criteria for a minimal risk study and approved a waiver for informed consent.

## Supporting information


**TABLE S1** Clinical characteristics at time of urinary catecholamine collection

## Data Availability

The data that support the findings of this study are available on reasonable request to the corresponding author. The data are not publicly available due to privacy or ethical restrictions.
