# Supplementary material for: Catecholamines in neuroblastoma: Driver of hypertension, or solely a marker of disease?
Source: Cancer Rep (Hoboken). 2021 Oct 6;5(8):e1569. doi: 10.1002/cnr2.1569 (PMC9351666; doi:10.1002/cnr2.1569)
Supplement: Supplementary file 1 — TABLE S1 Clinical characteristics at time of urinary catecholamine collection [file CNR2-5-e1569-s001.docx]

Supplemental Table S1. Clinical characteristics at time of urinary catecholamine collection

SBP: systolic blood pressure; DBP: diastolic blood pressure; ULRR: upper limit of the reference range

| Patient ID | Normetanephrine/creatinine (as ratio of ULRR) | Metanephrine/creatinine (as ratio of ULRR) | VMA/ creatinine (as ratio of ULRR) | HVA/creatinine (as ratio of ULRR) | Time of sampling (days post presentation) | Chemotherapy initiated at time of sampling | Tumor biopsied or surgically resected prior to time of sampling | SBP* percentile on day of sampling | DBP percentile on day of sampling |
| --- | --- | --- | --- | --- | --- | --- | --- | --- | --- |
| 1 | 2.9 | 0.4 | 2.7 | 1.4 | 4 | N | Y | >99 | >99 |
| 2 | 3.1 | not collected | 3.4 | 9.2 | 0 | N | N | >99 | >99 |
| 3 | 30.7 | 0.87 | 5.1 | 1.36 | 4 | N | N | >99 | - |
| 4 | 2.3 | 0.37 | 2.5 | 2.28 | 1 | N | N | >99 | >99 |
| 5 | 5.2 | not collected | 7.3 | 2.85 | 4 | N | N | >99 | >99 |
| 6 | 73.1 | not collected | 20.9 | 16.3 | 0 | N | N | >99 | 68 |
| 7 | 3.7 | 0.6 | 2.5 | 4.1 | 1 | N | N | >99 | >99 |
| 8 | 1.4 | 0 | 0.8 | 1.37 | 1 | N | N | 98 | >99 |
| 9 | 7.2 | 0 | 6.2 | 3.2 | 0 | N | N | >99 | 40 |
| 10 | 140.1 | 0.76 | 30.3 | 10.4 | 1 | N | N | >99 | >99 |

*Mean arterial pressure used in 1 patient (antenatal diagnosis)
